# Associations of cereal fiber intake with rheumatoid arthritis mediated by dietary inflammatory index: insights from NHANES 2011–2020

**DOI:** 10.1038/s41598-024-52806-w

**Published:** 2024-01-29

**Authors:** Huijuan Wan, Ya Zhang, Zhongxing Ning, Mingjiang Liu, Shudong Yang

**Affiliations:** 1 Department of Nephrology and Rheumatology, The Seconds Affiliated Hospital, Hengyang Medical School, University of South China, Hengyang, 421001 People’s Republic of China; 2 Department of Gland Surgery, The Affiliated Nanhua Hospital, Hengyang Medical School, University of South China, Hengyang, 421002 People’s Republic of China; 3 Guangxi Hospital Division of the First Affiliated Hospital, Sun Yat-Sen University, Guangzhou, People’s Republic of China; 4 Department of Hand and Microsurgery, Hengyang Medical School, The Affiliated Nanhua Hospital, University of South China, Hengyang, 421002 People’s Republic of China; 5 Department of Traumatic Orthopedics, The Seconds Affiliated Hospital, Hengyang Medical School, University of South China, No. 35 Jiefang Road, Zhengxiang District, Hengyang City, 421001 Hunan Province People’s Republic of China

**Keywords:** Health care, Medical research, Rheumatology

## Abstract

Rheumatoid Arthritis (RA) is an increasingly prevalent inflammatory disorder worldwide. Its complex etiology has recently brought dietary factors, particularly fiber intake, into focus as potential influencers. Our study investigates the intricate relationship between various sources of dietary fiber and RA, emphasizing the mediating role of the Dietary Inflammatory Index (DII). Leveraging data from the National Health and Nutrition Examination Survey spanning 2011 to 2020. We meticulously assessed dietary fiber intake through dual 24 h dietary recall interviews, while RA diagnoses were established based on comprehensive medical surveys. The relationships between fiber intake, RA prevalence, and DII mediation were analyzed using sophisticated multivariate logistic regression and mediation analysis. Among our study cohort, 7% were diagnosed with RA. We observed a notable inverse correlation between increased total fiber intake, particularly 5 g/day increments, and the incidence of RA, with cereal fiber intake emerging as the primary mitigating factor. Intriguingly, the DII played a significant role in mediating this association, especially regarding cereal fiber. Our findings reveal a significant association between higher cereal fiber consumption and a reduced prevalence of RA. Additionally, the DII stands out as a pivotal mediator in this relationship, highlighting dietary management's critical role in preventing and managing RA.

## Introduction

Rheumatoid arthritis (RA) is a globally prevalent inflammatory disorder characterized by symmetric inflammatory polyarthritis, leading to significant joint damage and functional impairment^1,2^. Since 1990, there has been a concerning 8.2% annual increase in RA cases worldwide, highlighting the growing need for effective detection and prevention strategies in an aging global population^3^. The etiology of RA is complex and not fully understood, but recent studies have started to shed light on dietary factors as potential contributors to its development^4^.

Recent scholarly work suggests that dietary fiber intake may have a therapeutic effect against inflammatory conditions, with the role of gut microbiota and its byproducts becoming increasingly recognized^5,6^. Previous research has pointed to the potential benefits of increased fiber intake in reducing systemic inflammation in individuals with RA^7^. A groundbreaking study utilizing data from the UK Biobank has uncovered the potential protective effects of a diet rich in dietary fiber and polyunsaturated fatty acids against the onset of RA. However, the specific impacts of various fiber sources, such as grains, vegetables, and fruits, on RA risk are still not fully explored.

Inflammation is a key factor in the development of RA, and the Dietary Inflammatory Index (DII) serves as a vital tool for assessing the overall inflammatory potential of a diet by considering the inflammatory properties of different dietary components. A growing number of studies highlight the significant role of DII in influencing RA susceptibility and progression^8^. Current theories suggest that a diet rich in dietary fiber can mitigate inflammation and thus reduce the risk of RA. However, there is still a gap in research regarding the extent and nature of the DII’s mediating role in the relationship between dietary fiber intake and RA risk.

Considering these factors, we undertook a cross-sectional study using data from the 2011–2020 National Health and Nutrition Examination Survey (NHANES). Our objective was dual: to explore the relationship between various sources of dietary fiber and RA risk and to elucidate the potential mediating role of the DII in the interaction between dietary fiber intake and the onset of RA.

## Methods

### Study population

**Figure 1:**
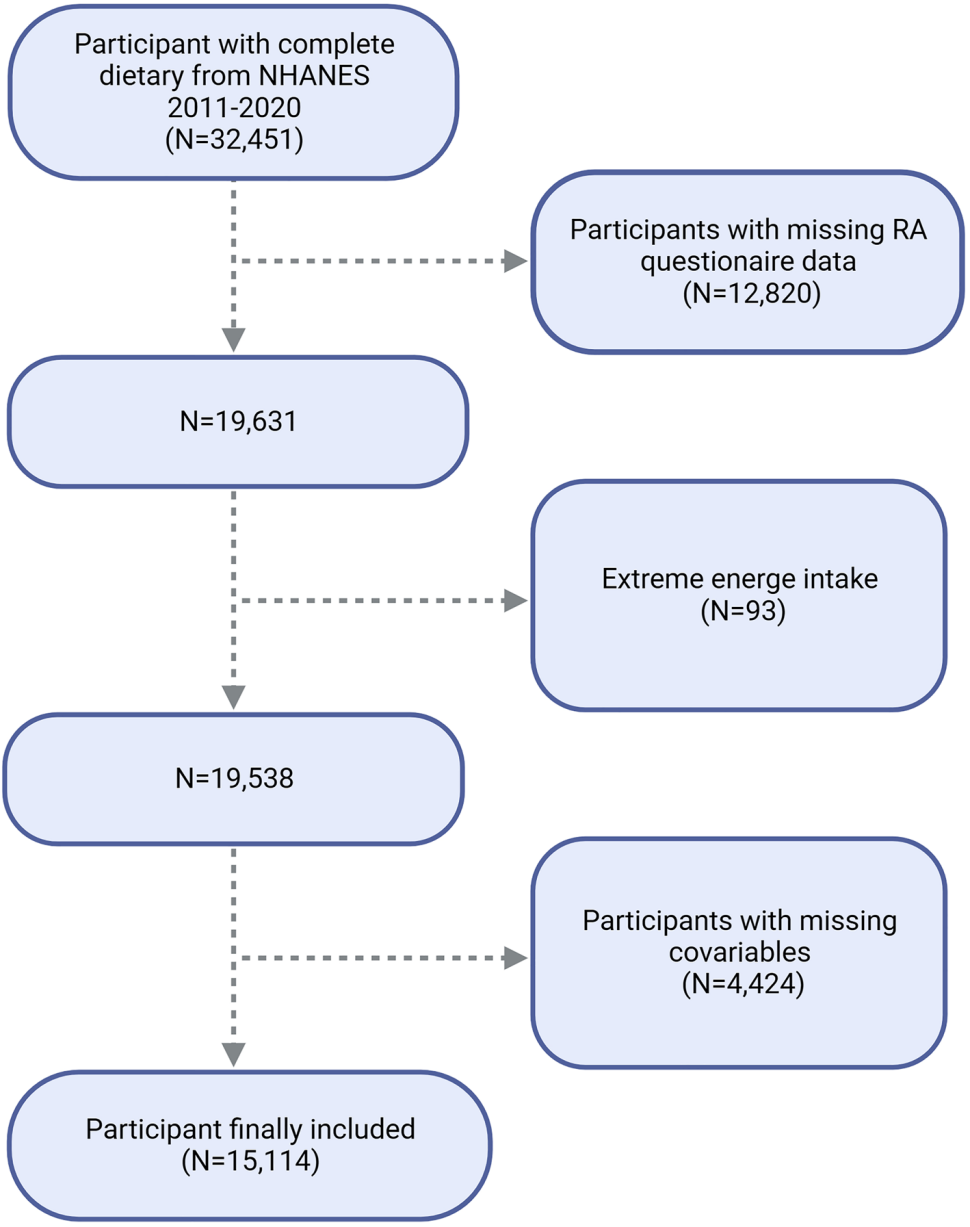
Flow chart of participants selection. NHANES, National Health and Nutrition Examination Survey; RA, Rheumatoid arthritis.

The National Center for Health Statistics (NCHS) conducted the NHANES, a nationally representative survey of the non-institutionalized population in the United States, as the basis for this study^9–11^. All study procedures were approved by the Research Ethics Review Board of NCHS, and informed consent was obtained from all participants. The present study complies with the guidelines for cross-sectional research provided by Strengthening the Reporting of Observational research in Epidemiology (STROBE). The dataset used in this study includes data from 2011 to 2020. In this study, after excluding participants without RA data (n = 12,820), and those with extreme total energy intakes (n = 93), a total of 15,114 samples were enrolled for the final analysis (Fig. 1).

We excluded participants who did not have self-reported RA data (n = 12,820), those with incomplete or missing dietary intake data (n = 13,011), those with missing covariate data (n = 4,424), and those with extreme total energy intakes (n = 93). The final sample for analysis included 15,114 participants (Fig. 1).

### Variables definition

**Figure 2:**
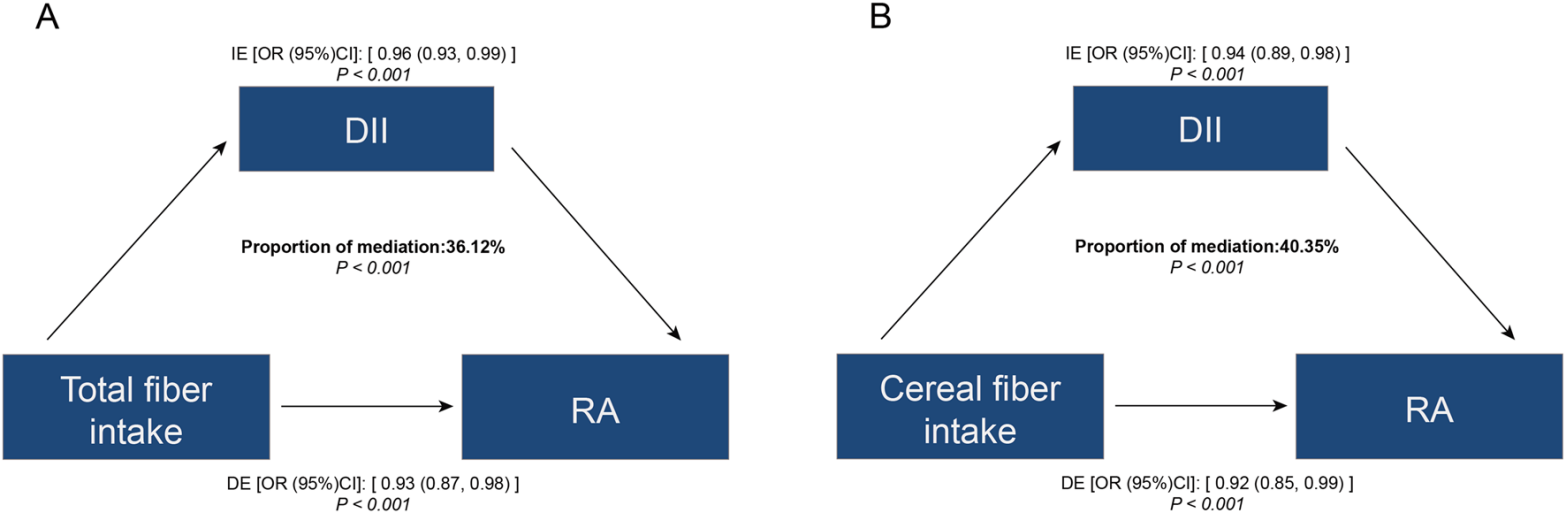
Estimated proportion of the association between fiber intake and RA mediated by DII. (**A**) Total fiber intake and RA incidence; (**B**) Cereal fiber intake and RA incidence. IE, Indirect effect; DE, Direct effect; mediation proportion = IE/DE + IE.

In alignment with prior research^12,13^, we enlisted a certified nutritionist to conduct two 24 h dietary recall sessions, assessing participants’ fiber intake. The initial session was conducted in person at the Mobile Examination Center (MEC), followed by a subsequent telephone session within 3 to 10 days. We calculated the average fiber consumption over these two days, adjusting for individual weight factors. Dietary fiber sources, including grains, vegetables, and fruits, were identified by collating relevant food codes (Fig. 2).

Professionals and two medical conditions accessed RA questionnaires to gather the data. When participants answered “yes” to the question, “Has your doctor ever told you to have arthritis,” we assumed these participants had arthritis. When this group of participants answered “rheumatoid arthritis” to the question “Which type of arthritis was it?”, we considered that these participants had RA. A meta-analysis that included 16 epidemiological studies demonstrated that self-reported RA is highly accurate and acceptable in large studies^13^.

The DII was employed as a critical metric to assess the inflammatory potential of various dietary components, ranging from vitamins to minerals^14^. We calculated the DII score by determining individual dietary markers and their corresponding z-scores, subsequently standardizing these into median percentiles. The standardized inflammatory effect of each percentile was then aggregated to form the final DII score for each participant. The selection of covariates for our analysis was informed by established links between fiber consumption, RA, and factors ranging from demographics to lifestyle habits.

Covariables were chosen based on prior knowledge as factors associated with fiber intake and RA. Covariates included age, gender, race, body mass index, family income-to-poverty ratio (PIR), menopause status (引用), moderate activities, waist circumference, energy intake, drinking alcohol status education level, and smoking status.

### Statistical analysis

R software (version 4.3.0) alongside Empowerstats (version 4.1) powered all statistical evaluations. Participant demographics were segmented by RA status and gauged using the chi-square and t-test. Advanced logistic regression procedures aimed to discern the connections between fiber consumption, inflammation markers, and RA prevalence^15–17^. To ascertain the potential intermediary role of DII between fiber consumption and RA emergence, we deployed a parallel mediator approach, using distinct indicators as mediators^18^. Herein, the direct effect (DE) portrays inflammation’s influence on RA without intermediary factors, while indirect effects (IE) elucidate the mediator-influenced relationship between inflammation and RA. Mediator proportions were derived by equating IE with the overall effect (TE).

### Ethical approval and consent to participate

The studies involving human participants were reviewed and approved by the NCHS Ethics Review Board. The patients/participants provided their written informed consent to participate in this study.

## Results

Our analysis encompassed 15,114 subjects, with an average age of 46.37 (16.89) years during the evaluation. This cohort identified 1053 participants (approximately 7%) with RA. The fiber consumption median for the RA group stood at 13.6 g/day, spanning from 0 to 97.4 g/day, whereas for the non-RA counterparts, it was 14.7 g/day, ranging from 0 to 134.8 g/day. When juxtaposed against the non-RA group, RA-afflicted individuals predominantly comprised females, exhibited advanced age, possessed inferior educational backgrounds, and reported reduced income levels. Lifestyle-wise, RA subjects showcased heightened tendencies for smoking and alcohol consumption and diminished engagement in physical activities. Physiologically, they registered increased BMI and waist measurements, while their dietary charts reflected reduced caloric and fiber intakes (Table 1). Demographic attributes were scrutinized using chi-square and t-test methodologies.

**Table 1:** Basic characteristics of participants by RA among U.S. adults.

Characteristics	RA (1053)	non-RA (14,061)	*P* value
Age (years)	60.51 ± 12.92	45.31 ± 16.67	< 0.001
Sex, n (%)			< 0.001
Male	445 (42.26)	7136 (50.75)	
Female	608 (57.74)	6925 (49.25)	
Race/ethnicity, n (%)			< 0.001
Non-hispanic white	374 (35.52)	4893 (34.80)	
Non-hispanic black	364 (34.57)	3400 (24.18)	
Mexican American	126 (11.97)	1933 (13.75)	
Other race/multiracial	189 (17.94)	3835 (27.27)	
Education level, n (%)			< 0.001
Less than high school	294 (27.92)	2528 (17.98)	
High school	262 (24.88)	3092 (21.99)	
More than high school	497 (47.20)	8441 (60.03)	
Moderate activities, n (%)			< 0.001
Yes	355 (33.71)	6266 (44.56)	
No	698 (66.29)	7795 (55.44)	
Smoking status, n (%)			< 0.001
Never	496 (47.10)	8635 (61.41)	
Ever	557 (52.90)	5426 (38.59)	
Drinking alcohol status, n (%)			< 0.001
Never	834 (79.20)	10,867 (77.28)	
Ever	219 (20.80)	3194 (22.72)	
Family PIR	2.11 ± 1.55	2.59 ± 1.65	< 0.001
BMI (kg/m^2^)	31.44 ± 7.92	28.93 ± 6.91	< 0.001
Waist circumference (cm)	105.65 ± 16.64	98.24 ± 16.38	< 0.001
Energy intake (kcal/day)	1817.14 ± 824.21	1979.06 ± 943.68	
Total fiber intake (g/day)	15.37 ± 9.84	17.12 ± 11.37	< 0.001
Cereal fiber intake (g/day)	8.30 ± 6.71	10.85 ± 7.54	< 0.001
Vegetable fiber intake (g/day)	3.98 ± 3.84	5.49 ± 5.64	< 0.001
Fruit fiber intake (g/day)	2.68 ± 2.93	3.70 ± 3.92	< 0.001
hs-CRP (mg/L)	4.34 ± 2.69	2.79 ± 2.03	< 0.001
DII score	3.26 ± 1.76	0.84 ± 1.89	< 0.001

Mean ± SD for continuous variables: the *P* value was calculated by the weighted linear regression model.

(%) for categorical variables: the *P* value was calculated by the weighted chi-square test.

*RA* rheumatoid arthritis, *Family PIR* the ratio of family income to poverty, *BMI* body mass index, *hs-CRP* high-Sensitivity C-Reactive Protein, *DII* dietary inflammatory index.

Table 2 elaborates on the multivariate logistic regression analysis outcomes, highlighting an inverse correlation between augmented total fiber intake (5 g/day) and RA emergence [0.89 (0.86, 0.98)]. We employed a sensitivity analysis to refine this exploration, categorizing fiber consumption into tertiles. Those in the topmost fiber consumption tertile experienced a 25% reduction in RA prevalence relative to their lowest tertile peers [0.75 (0.63, 0.88)]. We further delved into the influence of fiber sources on RA incidence. Post multi-variable adjustments, only cereal fiber intake emerged as significantly inversely correlated with RA onset [0.85 (0.76, 1.02)], this inverse link being more pronounced in sensitivity analyses [0.72 (0.61, 0.85)]. Conversely, vegetable and fruit fiber consumption displayed tenuous and inconclusive links with RA emergence.

**Table 2:** Results of the multivariate logistic regression analysis of association between fiber intake and RA.

Dietary fiber intake (5 g/day)	Model 1 [OR (95% CI)]	Model 2 [OR (95% CI)]	Model 3 [OR (95% CI)]
Total fiber intake	0.86 (0.81, 0.91)	0.89 (0.85, 0.96)	0.89 (0.86, 0.98)
Categories			
Tertile 1	1.0 (ref)	1.0 (ref)	1.0 (ref)
Tertile 2	0.85 (0.73, 0.99)	0.80 (0.69, 0.94)	0.85 (0.72, 0.99)
Tertile 3	0.73 (0.62, 0.85)	0.79 (0.67, 0.93)	0.75 (0.63, 0.88)
Cereal fiber intake	0.78 (0.71, 0.85)	0.82 (0.75, 0.99)	0.85 (0.76, 1.02)
Categories			
Tertile 1	1.0 (ref)	1.0 (ref)	1.0 (ref)
Tertile 2	0.75 (0.70, 0.86)	0.77 (0.56, 1.00)	0.83 (0.70, 0.98)
Tertile 3	0.74 (0.67, 0.82)	0.75 (0.61, 0.89)	0.72 (0.61, 0.85)
Vegetable fiber intake	0.96 (0.89, 1.02)	0.96 (0.86, 1.06)	0.98 (0.87, 1.10)
Fruit fiber intake	0.99 (0.91, 1.07)	1.04 (0.95, 1.13)	1.03 (0.96, 1.10)

Model 1: no covariates were adjusted. Model 2: age, gender, and race were adjusted. Model 3: age, gender, race, educational level, BMI, waist circumference, Energy intake, family income-to-poverty ratio, moderate activities, smoking status and drinking alcohol status were adjusted.

Table 3 delineates fiber consumption’s associations with DII. The weighted multivariate linear regression results underscored a significant inverse relationship between total fiber intake and both DII [− 2.82 (− 3.28, − 2.35)] and Hs-CRP [− 0.19 (− 0.32, − 0.07)]. Similar trends were observed for cereal and vegetable fiber intakes about DII. However, the relationship between fruit fiber intake and RA incidence remained inconclusive.

**Table 3:** Results of the multivariate linear regression analysis of association between fiber intake and inflammatory indicators.

Dietary fiber intake (5 g/day)	DII score [β (95%) CI]	Hs-CRP (mg/L) [β (95%) CI]
Total fiber intake	– 2.82 (– 3.28, – 2.35)	– 0.19 (– 0.32, – 0.07)
Cereal fiber intake	– 3.56 (– 4.82, – 2.30)	– 0.23 (– 0.45, – 0.02)
Vegetable fiber intake	– 0.28 (– 0.52, – 0.03)	– 0.05 (– 0.13, 0.02)
Fruit fiber intake	0.03 (– 0.28, 0.32)	0.01 (– 0.10, 0.09)

Age, gender, race, educational level, BMI, waist circumference, Energy intake, family income-to-poverty ratio, moderate activities, smoking status and drinking alcohol status were adjusted.

Table 4 elucidates the multivariate logistic regression analysis insights into the DII-RA incidence relationship. A notable positive correlation surfaced between DII and RA prevalence, indicating a 26% surge in RA cases for each unit increment in DII [1.26 (1.01, 1.58)]. Moreover, participants with the highest DII readings exhibited a 62% amplification in RA incidence relative to the lowest DII group [1.62 (1.02, 2.18)].

**Table 4:** Results of the multivariate logistic regression analysis of association between inflammatory indicators and RA.

Exposure	Model 1 [OR (95% CI)]	Model 2 [OR (95% CI)]	Model 3 [OR (95% CI)]
DII score	1.32 (1.14, 1.62)	1.30 (1.13, 1.59)	1.26 (1.01, 1.58)
Categories			
Tertile 1	1.0 (ref)	1.0 (ref)	1.0 (ref)
Tertile 2	1.42 (1.11, 1.82)	1.32 (1.03, 1.62)	1.25 (0.92, 1.56)
Tertile 3	1.91 (1.22, 2.36)	1.88 (1.25, 2.17)	1.62 (1.02, 2.18)

Model 1: no covariates were adjusted. Model 2: age, gender, and race were adjusted. Model 3: age, gender, race, educational level, BMI, waist circumference, Energy intake, family income-to-poverty ratio, moderate activities, smoking status and drinking alcohol status were adjusted.

Our findings reveal that the DII plays a significant, albeit not predominant, mediating role in the association between total and cereal fiber intakes and RA incidence. Specifically, the mediation percentages of DII were found to be approximately 36.12% for total fiber intake and 40.35% for cereal fiber intake (*P* < 0.01), indicating a notable but not exclusive influence in this association (Fig. 2).

## Discussion

This study unveils two critical insights into the dietary habits of the American adult population. Firstly, it establishes a clear correlation between suboptimal fiber intake and an increased prevalence of RA, identifying cereal fiber as the primary mitigating factor in this inverse relationship. Secondly, our analysis highlights the DII as a critical intermediary in the association between fiber consumption and the onset of RA. Notably, while the DII’s mediating role falls below 50%, its direct impact considerably surpasses the indirect effect, emphasizing its integral role in the fiber-RA incidence connection.

An expanding reservoir of scientific literature is emphasizing the influence of dietary elements and habits on RA onset^19^. Diet, as a pivotal external determinant, can influence RA’s development by modulating gut microbiota, antigenic expression, as well as the body’s inflammatory and antioxidant defense systems^20,21^. Typically, RA patients are counseled to augment their intake of anti-inflammatory nutrients^22^, such as dietary fiber and specific polyunsaturated fatty acids^23^, which harbor both anti-inflammatory and antioxidant attributes, potentially arresting inflammation escalation^24^. Regrettably, the present dietary quality among RA patients falls short, with our findings revealing that such patients presented markedly elevated DII scores compared to the general participants. Even though nutritional guidelines advocate a surge in fiber intake, the majority of Americans fall short, consuming less than half of the suggested daily fiber quota, with RA patients faring even worse^25^. An inferior diet correlates with the longevity and intensity of RA manifestations, potentially amplifying RA onset risks^21,26^. Our data underscore the significance of the fiber’s origin, pinpointing cereal fiber as the prime contender in inflammation reduction. While prior research has postulated anti-inflammatory roles for fiber, drawing from its ability to satiate, enhance gut health, modify dietary regimes, and optimize lipid and glucose metabolism^27–29^, the unique significance of cereal fiber over its vegetable or fruit counterparts remains a puzzle, warranting deeper probing.

Historically, heightened fiber consumption was touted as a remedy for alleviating RA symptoms^30^. Yet, the nexus between dietary fiber and RA vulnerability remains enigmatic and contentious, with scant research probing this connection. Contradictory findings, like those from the EPIC-Norfolk study linking reduced fruit and vegetable intake with escalated inflammatory polyarthritis^31^ or the Greek case-control study associating cooked vegetable and olive oil consumption with reduced RA risks^32^, further muddle the picture. Amidst such ambiguity, our study presents a robust framework, leveraging a comprehensive dataset and stringent control mechanisms, thereby bolstering the credibility and consistency of its conclusions.

The complex interplay between dietary fiber consumption and the onset of RA continues to be an area of active research. The prevailing consensus suggests that inflammation is at the heart of this relationship. Diets rich in fiber, like the Mediterranean and vegan diets, have been shown to reduce inflammation in RA patients^30,33^. Large-scale observational studies further reinforce the connection between fiber intake and inflammatory markers in diverse populations. The gut-joint axis, particularly the anti-inflammatory effects of fiber fermentation on gut microbiota, offers a plausible explanation for these findings^34–36^. Gut dysbiosis has been identified as a key factor in the development of RA, both in animal models and human studies^37^. Gut dysbiosis, a potential disruptor of intestinal barrier functionality, has emerged as a cornerstone of RA’s genesis in mice and human studies^38–40^. Our mediation analysis underscores the inverse association between high cereal fiber intake and RA prevalence, establishing DII as a critical mediator in this relationship.

However, our study’s strengths significantly contribute to its value. A key strength is the incorporation of the DII, a novel approach in RA research. The DII provided a comprehensive measure of the inflammatory potential of participants’ diets, allowing for a nuanced analysis of how dietary components, particularly fiber, relate to RA incidence. This incorporation presents a more holistic understanding of diet’s role in RA, beyond the traditional focus on single nutrients or food groups. Another noteworthy aspect of our study is its in-depth examination of the relationship between various sources of dietary fiber and RA incidence. By distinguishing between different fiber sources, such as cereal, vegetable, and fruit fibers, our study offers a more detailed understanding of how specific dietary choices might influence RA risk. This is particularly relevant given the diverse roles of different fiber types in modulating inflammation and gut microbiota.

In reflecting upon the methodological aspects of our study, we recognize certain limitations inherent in the tools we employed. Concerning the DII, while it offers a comprehensive assessment of the diet’s inflammatory potential, it is important to note that many dietary components influence the DII^41^. This complexity necessitates a cautious interpretation when attributing the effects of specific nutritional elements, such as cereal fiber, to the DII. It is plausible that there are other contributing dietary factors that our analysis may not have fully accounted for. Moreover, the use of the 24 h food recall method in our study, although a widely accepted tool in dietary research, presents its own set of limitations. One significant concern is its potential inability to accurately capture habitual dietary patterns, considering the day-to-day variations in food intake. Additionally, the reliability of this method is contingent upon the participant’s ability to accurately recall and report their food consumption, which introduces the possibility of under or over-reporting^42,43^. Acknowledging these limitations, future research endeavors might benefit from employing multiple food recalls or food frequency questionnaires to glean a more representative and comprehensive picture of long-term dietary habits. Despite these limitations, the extensive dataset, meticulous approach to dietary intake data collection, and innovative use of the DII in our study make a valuable contribution to the literature on the interplay between dietary fiber, inflammation, and RA onset. Our findings provide a foundation for future research and potential nutritional interventions to manage or reduce the risk of RA.

## Conclusion

Our findings reveal a significant association between increased cereal fiber intake and a reduced prevalence of RA. This relationship is notably absent with vegetable or fruit fiber intake. Furthermore, the DII emerges as a crucial intermediary, elucidating the connection between higher cereal fiber consumption and lower RA occurrence.

## Data Availability

The survey data are publicly available on the internet for data users and researchers throughout the world ( www.cdc.gov/nchs/nhanes/).

## Abbreviations

RA: Rheumatoid arthritis
NHANES: National health and nutrition examination survey
NCHS: National center for health statistics
Hs-CRP: High-sensitivity C-reactive protein
DII: Dietary inflammatory index
DE: Direct effect
IE: Indirect effect
TE: Total effect
